# Effectiveness of a universal health-promoting parenting program: a randomized waitlist-controlled trial of All Children in Focus

**DOI:** 10.1186/1471-2458-14-1083

**Published:** 2014-10-18

**Authors:** Malin Ulfsdotter, Pia Enebrink, Lene Lindberg

**Affiliations:** Department of Clinical Neuroscience, Karolinska Institutet, Stockholm, Sweden; Department of Public Health Sciences, Karolinska Institutet, Stockholm, Sweden

**Keywords:** ABC program, Child health and development, Health promotion, Parenting program, Parental self-efficacy, Randomized controlled trial, Universal

## Abstract

**Background:**

Parenting programs have been highlighted as a way of supporting and empowering parents. As programs designed to promote children’s health and well-being are scarce, a new health-promotion program, All Children in Focus, has been developed. The purpose of this trial was to evaluate the potential effectiveness of the program in promoting parental self-efficacy and child health and development, as well as to investigate possible moderators of these outcomes.

**Methods:**

A multicenter randomized waitlist-controlled trial was conducted. The trial included 621 parents with children aged 3–12 years. Parents were randomized to receive the intervention directly or to join a waitlist control group. Parents completed questionnaires at baseline, 2 weeks after the intervention, and 6 months post-baseline. To evaluate potential effects of the program, as well as any moderating variables, multilevel modeling with a repeated-measures design was applied.

**Results:**

Parents in the intervention group reported that their self-efficacy (*p* < .001), as well as their perceptions of children’s health and development (*p* < .05), increased 6 months post-baseline when compared with parents in the control group. One variable was found to moderate both outcomes: parents’ positive mental health. Furthermore, parents’ educational level and number of children moderated parental self-efficacy, while the children’s age moderated child health and development. Having a poor positive mental health, a university-level education, more than one child in the family, and older children, made the families benefit more.

**Conclusions:**

In the first randomized controlled trial of All Children in Focus, we found that the program appears to promote both parental self-efficacy and children’s health and development in a general population. Additionally, we found that families may benefit differently depending on their baseline characteristics. This contributes to an existing understanding of the advantages of offering universal parenting programs as a public health approach to strengthening families**.** However, further research is needed to investigate long-term effects and mediating variables, as well as the potential cost-effectiveness of the program.

**Trial registration:**

Current Controlled Trials: ISRCTN70202532. November 7th 2012.

## Background

Children usually spend a great amount of time with their parents. Parents and parenting are therefore essential to target within the field of health promotion and prevention of mental health problems for children [1, 2]. One way to support parents is to offer participation in parent group programs [1]. Parenting programs generally aim to establish desirable change in parental behavior and thereby reduce problematic child behavior [3]. These programs are usually, according to the classification of prevention, divided into universal, selective, and indicated levels [4]. Universal programs are offered to everybody, selective programs to groups with a common risk factor, and indicated programs to individuals with identified problems. To date, the majority of studies on the effectiveness of parenting programs have focused on selective and indicated programs. Reviews have shown that these programs have the potential to improve parenting skills and decrease behavioral problems in children [5–9].

Evaluations of universally offered parenting programs have emerged more rapidly in the last decade. One example of an evaluated universal program is the International Child Development Programme (ICDP), which includes eight sessions. The program was found to improve parents’ strategies and attitudes toward child management. The parents also reported experiencing less impact from child difficulties [10]. Another example of a universally offered program is a shortened version (six sessions) of the Incredible Years program. An evaluation by Reedtz and colleagues [11] demonstrated that harsh parenting decreased, positive parenting and the parents’ sense of competence were strengthened, and child behavior problems were reduced after parents participated in the program. Morawska and colleagues [12] reported reduced child behavior problems, improved parenting styles, and parental self-efficacy from a brief universal parenting discussion group (one session and two follow-up telephone consultations). Hiscock and colleagues [13] described how mothers were less likely to report harsh parenting and unreasonable expectations of child development when a short program (three sessions) was offered universally. Evaluations of universally offered Triple-P parent groups (four sessions followed by phone support) showed reductions in dysfunctional parenting and child behavior problems [14, 15] and positive effects on parent mental health and child-rearing conflicts [15]. In contrast to these positive findings, other evaluations did not report any effectiveness of programs offered at a universal level [3, 16]. In the cited evaluations of universally offered parenting programs, parents were recruited and groups were run at child health services, health centers, preschools, and schools. Only a few of the evaluations contained information regarding the socioeconomic status (SES) of the area where the program was implemented. Simkiss and colleagues [16] recruited parents in deprived areas, while Hahlweg and colleagues [14] reported that parents living in high SES areas participated in the study to a greater extent than parents living in low or medium SES areas.

It is commonly accepted that essential principles of positive parenting are cross-culturally robust [17]. When a program is newly developed, however, it may be relevant to clarify whether a program works for all parents, regardless of country of origin. Child age and gender, as well as parental SES and depression, have been found to moderate the outcome in previous studies of parenting programs [10, 18]. Furthermore, the number of children in the family has been associated positively with parental outcomes [19], and could theoretically be a moderator of outcomes in parenting programs. Except for the study by Sherr and colleagues [10], existing knowledge regarding moderating variables seems to be based on selective and indicated parenting programs, whereas for universal parenting interventions there is a lack of knowledge regarding potential moderators.

A public health approach has been suggested by Sanders [17] to ensure that more parents are being offered parenting programs, which thereby enables significant public health benefits. Within this approach, the universal programs would play a fundamental role. However, universal programs originally developed for certain clinical groups may not be appropriate for a generally healthy population of parents with everyday challenges [10]. For the programs to appeal to parents in the general population, an appropriate complement to the regular universal programs that aim to prevent child problems might be programs emphasizing the promotion of health. An intervention focused on health promotion aims to increase positive outcomes [20]. Offering programs on a universal basis could also contribute to less stigmatization regarding participation in parenting programs, as inclusion is not based on experiencing problems or deficits.

### The ABC program and study objective

Universal health-promoting parenting programs appear to be rather uncommon. To bridge this gap, a brief (four-session) program, All Children in Focus (the ABC program), was developed. The program includes all parents with children aged 3–12 years. The ABC targets the parent–child relationship, as well as parental everyday experiences, and aims to promote children’s development. Even though the ABC program itself is not intended to reduce problematic child behavior, it consists of components shown to be effective in programs for the prevention or treatment of child behavior problems [21, 22]. The components were chosen due to the lack of knowledge about evidence-based components in universal programs and were adapted to fit a health-promotion perspective.

The ABC program is similar to other universally offered programs in the sense that it is theory-based and organized as group meetings. The group leaders follow a manual, and parents are provided with parenting strategies. However, several other universally offered programs are developed to prevent or reduce child behavior problems [3, 11, 12, 14, 15], while the ABC program was developed to promote children’s development. Regarding program content, ABC is most similar to ICDP [10] and the Family Links Nurturing Programme (FLNP) [16]. There are similarities in the aim of empowering parents, with group leaders working as facilitators and encouraging parents to try approaches. In addition, the content is focused on promoting factors, such as positive attention and empathy [10, 16]. ICDP and FLNP are more intensive, however, as they include double the amount of sessions or more [10, 16]. There are also similarities to Triple-P [14]. One of the aims of Triple-P is to promote children’s competence and development, with strategies such as quality time and praise. Triple-P also aims to help parents manage misbehavior with strategies such as planned ignorance and time-out [14], characteristics which differentiate it from ABC. The content of other programs involve child noncompliance [12], as well as parental risk factors for child behavior problems, such as unreasonable expectations [13], which are also different from the content of ABC. However, what distinguishes the ABC program the most from other universally offered programs is possibly the fact that ABC consists of evidence-based components [21, 22].

A pilot study of the ABC program showed improvements in child mental and physical health and independence/autonomy, as well as in parental self-efficacy, empathy, and ability to provide guidance for the child [23]. However, as the study was a preliminary investigation of the ABC program, it lacked a control group. To evaluate the potential effects of the universal health-promoting program ABC more rigorously, a randomized controlled trial was planned.

The overall objective of the present study was to evaluate the effectiveness of the ABC program in a randomized waitlist-controlled trial. First, we investigated the effectiveness of the program in promoting parental self-efficacy and child health and development. Second, we tested the impact of potential moderators on the outcome variables. Investigated moderators included the child’s gender and age, the parent’s country of birth, educational level, and positive mental health, and the number of children in the family.

## Methods

The methods section gives an overview of the trial. For a detailed description, please see the study protocol [24].

### Trial design

The ABC program was evaluated in a multicenter randomized waitlist-controlled trial, where parents were randomized to either (a) receive the ABC program directly or (b) join a waitlist-control group receiving the intervention after approximately six months. All parents completed a pre-measurement questionnaire (at baseline), a questionnaire two weeks after the intervention (post-measurement), and a questionnaire six months post-baseline (follow-up measurement). Ethical approval for the trial was obtained from the Regional Ethical Review Board in Stockholm (Dnr: 2012/93-31/5).

### Participants, randomization, and setting

**Figure 1:**
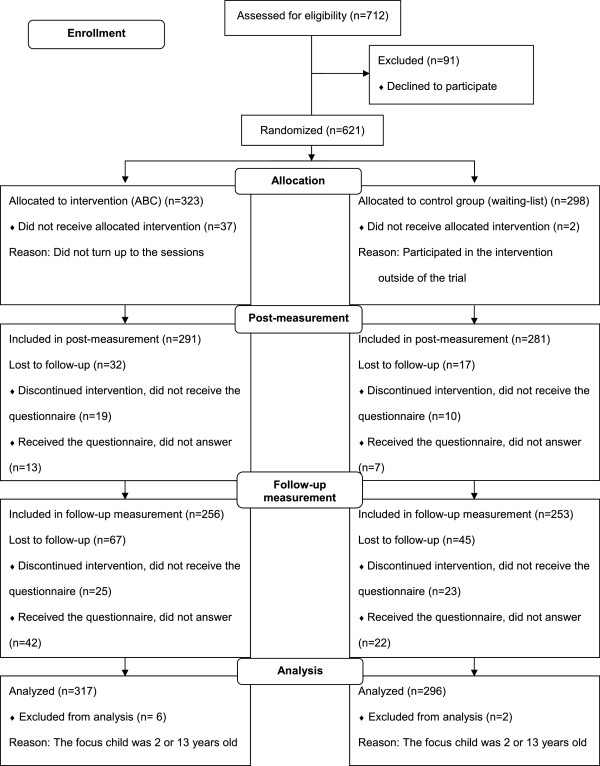
**CONSORT flowchart showing the enrollment of parents in the trial of ABC.**

Parents with children aged 3–12 were recruited to the trial during two waves, spring (February–March) and fall (September–October), in 2012. During the recruitment phase, all parents were invited to an informational meeting on local premises. Parents were informed orally about the trial by research staff, while trained group leaders informed them about the ABC program. They received written information about the trial, and those interested in participating signed an informed consent. All parents who agreed to participate completed the pre-measure assessment (a questionnaire). The intention was to include 300 parents each in the intervention and control groups, according to a sample-size calculation. Parents were randomized by the researchers at the individual level, at a ratio of 1:1. Randomization was performed for each municipality/city district using the random-sampling function in IBM SPSS Statistics for Windows, Version 20 (IBM Corp, Armonk, NY). Couples were randomized as a single unit. In total, 621 parents were recruited to the trial. See the flowchart (Figure 1) for details on the enrollment of the parents.

Recruitment of parents and implementation of parent groups were conducted locally in 11 municipalities and city districts in the County of Stockholm, Sweden. The settings for recruitment were maternity health services, child health services, preschools, and schools. Strategies for recruiting parents included: advertising in the local press and on websites, contacting parents personally, sending letters to parents, and showing a specially produced ABC promotional video at local supermarkets. The most common settings for hosting the ABC groups were schools and preschools; however, family centers and other community facilities were also frequently used.

### Measures

Parental questionnaires were used to assess parental self-efficacy, child health and development, and the baseline characteristics of the study population.

*Parental Self-Efficacy* (PSE) was measured using a 48-item questionnaire. The development of this questionnaire was influenced by another measure, Tool to Measure Parental Self-Efficacy (TOPSE) [25]. The measure comprises eight subscales: positive emotion, being with your child, empathy, guiding, rules, pressures, acceptance, and experience. Parents rated statements such as, “I can show my child affection” and “I keep calm when my child misbehaves” on an 11-point Likert scale, ranging from 0 (*completely disagree*) to 10 (*totally agree*). This resulted in a total score between 0 and 480, where a higher score was equivalent to a higher level of PSE. The construct validity of PSE was explored via confirmatory factor analysis. The analysis showed that the fit of the model to the data was acceptable (RMSEA = .072). The internal reliabilities (Cronbach’s alpha) for the full PSE scale in the present trial were .94 at baseline, .93 at post-measurement, and .94 at follow-up measurement.

*Child Health and Development* (CHD) was measured using a 35-item questionnaire. The development of CHD was based on an established health-related quality of life instrument [26] and was tested on data from previous pilot studies of the ABC program (n =405). The questionnaire measures parents’ perceptions of their child’s physical and mental health, emotional development, independence, family relations, and social competence. Parents rated questions such as, “How would your child describe (s)he is feeling in general?” on a 5-point scale, yielding a total score between 35 and 175, where a higher score was equivalent to better child health status. The CHD was validated using pilot-study data and baseline data from the present trial. The confirmatory factor analysis showed that the fit of the model was acceptable (RMSEA = .074). Internal reliabilities in this trial were .93 for the complete scale at baseline, .92 at post-measurement, and .92 at follow-up measurement.

The questionnaire also included questions about the age and gender of the focus child, as well as the parent’s country of birth (i.e., born in Sweden/not born in Sweden), educational level (i.e., university-level/not university level), positive mental health, and number of children in the family (i.e., one child/several children). These variables were included in the study to test for moderators. Parent’s mental health was measured using the General Health Questionnaire (GHQ) [27], where the six positively phrased questions were used to measure positive mental health [28]. An example of a positively phrased question was, “Have you been feeling reasonable happy, all things considered?” [28]. Each of the six items was rated on a four-point Likert scale (0–3), resulting in a total score ranging from 0–18. A higher score was equivalent to higher positive mental health.

### Intervention

ABC consisted of four 2.5-hour structured sessions given to parents every other week. Components included in the sessions were: positive attention and warmth, parent–child time and child-directed play, positive parenting strategies, and consistent parenting. The sessions consisted of discussions and short films, while role-plays exercises were used to facilitate the discussions. Each ABC group was run by two trained group leaders, and groups within the trial contained seven parents on average. After approximately 2–3 months, a booster session was offered to parents; during the trial, this was offered after the six-month post-baseline measurement. The booster session included review from the four previous sessions, plus an introduction to one of three new topics (e.g., “boys and girls,” “sibling relations,” “teens”). The program targeted an important protective factor for children, namely, the parent–child relationship [1], and aimed to promote children’s development. The ABC program has been described thoroughly in other sources [24].

### Statistical analyses

Chi-square tests and t-tests for independent groups were performed to examine baseline differences between the intervention and control groups. The same tests were also used to examine differences between parents who did not complete the post- and follow-up measurements and parents who did.

Multilevel linear modeling (MLM) with a repeated-measures design was used (SPSS, mixed models) to evaluate the effectiveness of the program. There are several advantages to MLM, compared with other analyses, such as the lack of requirement for complete data across time points and the possibility to test individual differences in growth curves [29]. In a repeated-measures study design, individual scores are nested within individuals and might in turn be nested within, for example, households [30]. Nested data are more likely to correlate within the group. For example, responses from parents in the same family unit are more likely to correlate highly, compared with responses by parents in general.

A three-level model was run to account for the 621 individuals being nested within three measurements, and that 220 of the included individuals were nested in 110 households. Time-related variables were constructed to manage the nonlinear growth trajectories in both primary outcome measures. There are different ways to code the time-related variable when a growth trajectory is found to be nonlinear. One way is to use a quadratic time variable (where the three measurement points, i.e. baseline-, post-, and follow-up measurement, are coded as 0, 1, 4), which captures any fluctuations in the rate of change across time points [31]. Another way is to code the first measurement occasion (the baseline) as 0 and the third measurement occasion (the follow-up) as 1. The growth that occurs over the entire trend is captured when using this approach [31]. The code for the second measurement point (the post-measurement), is in this approach, found by generating a variety of specifications and is determined by the best model fit [31]. The second measurement point is an intermediate point of the growth trajectory, and the code shows if the growth trajectory is close to being linear (i.e., if it is close to .5). Both the quadratic time-variable approach and 0–1 approach were tested on our data. The approach of coding the time variables as 0–1 gave the best model fit and was therefore selected. The three measurement points were coded as 0, .95, and 1 for PSE, while CHD was coded as 0, .85, and 1. The model included an interaction between time and condition. The intercept and time-related variables were used as random effects in the models, and best model fit was achieved with unstructured covariance type.

Effect sizes (η^2^) were calculated by subtracting the residual variance of the larger model from the residual variance of the intercept model, and then dividing the sum with the residual variance of the intercept model (using models that did not include random effects of time) [29]. Adopting the guidelines of Cohen [32], .02 represents a small effect size, .13 a medium effect size, and .26 a large effect size.

Another advantage of MLM is that the model can be adjusted simultaneously for the effects of numerous covariates. Several MLM analyses were run to evaluate the potential moderating effects, including interactions between condition, time, and the potential moderator (e.g., child age and gender, parent’s country of birth, educational level, positive mental health, number of children in the family). As a second step, all significant interactions were included in a final model, with scaled identity used as covariance type. For the moderation analyses, the variables of child age and positive mental health were centered on the grand-mean.

Intention-to-treat analyses were conducted, and IBM SPSS Statistics for Windows, Version 22 (IBM Corp, Armonk, NY) was used for all statistical analyses. The alpha level was set to < .05.

## Results

### Attrition and participation

Of the participating parents, 572 (92%) completed the post-measurement, and 509 (82%) the follow-up measurement (see Figure 1). The attrition rate was significantly higher in the intervention group compared with the control group at post-measurement (*χ*^2^[1] =3.94, *p* = .047), whereas the difference was not significant at follow-up (*χ*^2^[1] =3.61, *p* = .057).

Compared with the intervention-group parents who completed the post-measurement assessment, intervention-group parents who did not complete the assessment rated parental self-efficacy (*t*[305] =2.07, *p* = .040), as well as child health and development (*t*[277] =2.50, *p* = .013) higher at baseline. Additionally, intervention-group parents who did not complete the post-measurement also participated in fewer ABC sessions (*t*[35.7] =9.85, *p* < .001), compared with intervention-group parents who did complete the measurement.

When comparing parents who failed to complete the post-measurement with parents who completed the measurement, the former were younger (*t*[611] =2.06, *p* = .04), reported a lower family income (*t*[586] =2.45, *p* = .014), and were more often born outside of Sweden (*χ*^2^[1] =7.29, *p* = .007). The former group also had younger children that were the focus of the ABC (*t*[611] =2.15, *p* = .032) and rated their child’s health and development more positively (*t*[544] =2.02, *p* = .044). Parents who failed to complete the follow-up assessment rated their positive mental health more highly (*t*[603] = -2.61, *p* = .009) and were more often born outside of Sweden (*χ*^2^[1] =22.29, *p* < .001), compared with parents who completed the follow-up measurement. Regarding other baseline characteristics and outcome measures, there were no differences between parents who failed to complete the measurements and parents who completed the measurements.

Of the 323 parents randomized to the intervention group, 37 (11.5%) did not attend the ABC program at all, whereas 170 parents (52.6%) participated in all four sessions, 83 (25.7%) in three sessions, 28 (8.7%) in two sessions, and 5 parents (1.5%) participated in only one session.

### Comparisons of intervention and control groups at baseline

No differences were found in baseline characteristics or the outcome measures at baseline, when comparing the intervention and control groups. See Table 1 for a detailed description.

**Table 1:** **Baseline characteristics for the intervention and control groups, reported as mean (standard deviation) or number (percent)**

Variable	Intervention (*n*=317)	Control (*n*=296)	Statistics	*p*-value
**Child characteristics**				
Age of focus child (years)	6.09 (2.6)	6.26 (2.6)	*t*(611) = -0.79	.432
Gender (boys)	181 (57.3)	168 (56.8)	*χ* ^2^(1) =0.017	.896
**Parent and family characteristics**				
Age (years)	38.09 (5.5)	38.38 (5.4)	*t*(611) = -0.65	.517
Gender (women)	238 (75.1)	211 (71.3)	*χ* ^2^(1) =1.13	.289
Born in Sweden	249 (78.5)	222 (75.0)	*χ* ^2^(1) =1.08	.298
Single-parent households	32 (10.2)	28 (9.5)	*χ* ^2^(1) =0.08	.782
Single-child households	50 (15.8)	45 (15.2)	*χ* ^2^(1) =0.04	.845
Higher education^a^	176 (55.5)	177 (60.2)	*χ* ^2^(1) =1.37	.242
Family income^b^	55108 (22481)	61484 (51421)	*t*(382) = -1.93	.055
Parental Self-Efficacy	363.8 (51.6)	366.8 (48.5)	*t*(593) = -0.73	.467
Child Health & Development	137.2 (13.4)	137.0 (14.6)	*t*(544) =0.17	.865
General Health Questionnaire	11.7 (2.7)	11.6 (2.5)	*t*(603) = -0.35	.729

^a^Higher education is defined as having completed a university education.

^b^Income is the monthly family income in SEK before taxation (SEK = Swedish krona, 1 Euro =9.23 SEK [September 2014]).

### Program effectiveness

Concerning PSE, there was an interaction effect representing an increase in the intervention group across the entire measurement period. Parents in the intervention group reported that their self-efficacy increased after they had attended the ABC program, compared with parents in the control group. The effect for the intervention group across the measurement period was an estimated increase of 24.1 points (95% CI =20.25, 27.99) in the total PSE score, representing a moderate η^2^ effect size of .18.

We also found an interaction effect with CHD, implying an increase in the intervention group across the measurement period. Parents in the intervention group rated their children’s health and development higher after they had attended the program, compared with parents in the control group. The effect for the intervention group, across the measurement period, was an estimated improvement of 6.7 points (95% CI =5.32, 8.10) in the total CHD score, representing a moderate effect size of .15 (η^2^). For further details, see Table 2.

**Table 2:** **Mixed linear model estimates for Parental Self-Efficacy (PSE) and Child Health and Development (CHD)**

Variable	β	SE	*t*	95% CI	*p*-value	η ^2^
**PSE**						
Intercept	365.17	2.94	124.27	359.40, 370.95	.000	
Time	24.12	1.97	12.24	20.25, 27.99	.000	
Group (C)	2.49	4.24	0.59	-5.83, 10.82	.557	
Time × Group (C)	-15.24	2.82	-5.41	-20.78, -9.71	.000	.18
**CHD**						
Intercept	137.05	0.86	159.43	135.36, 138.74	.000	
Time	6.71	0.71	9.49	5.32, 8.10	.000	
Group (C)	-0.20	1.24	-0.16	-2.63, 2.23	.874	
Time × Group (C)	-2.21	1.01	-2.18	-4.20, -0.21	.030	.15

Note. *(C)* = Control group.

### Moderating effects on parental self-efficacy

Regarding the outcome variable of PSE (Table 3), three-way interaction effects were found for parents’ educational level, positive mental health, and number of children. Parents in the intervention group with university-level education showed a greater increase in self-efficacy over time, compared with parents without university education. Furthermore, parents in the intervention group who reported higher positive mental health showed a smaller increase in self-efficacy over time, compared with parents who reported lower positive mental health. The effect of the number of children, in this case, having more than one child, was positive over time—that is, parents in the intervention group with more than one child experienced a greater increase in self-efficacy over time, compared with parents who had only one child. These patterns were not evident for the control group. The other investigated variables, i.e., age and gender of the children, as well as parents’ country of birth, did not moderate the PSE outcome over time.

**Table 3:** **Mixed linear model estimates for moderating effects on Parental Self-Efficacy (PSE)**

Variables	β	SE	*t*	95% CI	*p*-value
Child age × Time × (I)	-0.55	0.69	-0.80	-1.90, 0.80	.423
Child age × Time × (C)	.11	0.71	0.16	-1.29, 1.52	.874
Child gender(boy) × Time × (I)	.81	3.70	0.22	-6.44, 8.07	.826
Child gender(boy) × Time × (C)	2.65	3.74	0.71	-4.70, 10.00	.479
Parents’ birth country (foreign-born) × Time × (I)	-4.39	4.53	-0.97	-13.28, 4.50	.333
Parents’ birth country (foreign-born) × Time × (C)	-2.45	4.44	-0.55	-11.17, 6.26	.580
Parental education (university) × Time × (I)	9.44	3.67	2.57	2.23, 16.65	.010
Parental education (university) × Time × (C)	2.87	3.72	0.77	-4.44, 10.17	.442
Parental positive mental health × Time × (I)	-2.13	0.68	-3.13	-3.46, -0.79	.002
Parental positive mental health × Time × (C)	0.07	0.70	0.10	-1.31, 1.45	.920
Number of children^a^ × Time × (I)	16.17	5.24	3.09	5.88, 26.48	.002
Number of children^a^ × Time × (C)	-3.80	5.06	-0.75	-13.73, 6.14	.453

Note. *(I)* = Intervention group; *(C)* = Control group.

^a^Having more than one child.

### Moderating effects on child health and development

As seen in Table 4, three-way interaction effects were found for children’s age and parents’ positive mental health. With increased child age, there was a greater change over time in perceptions of children’s health and development in the intervention group. For parents in the intervention group reporting higher positive mental health, there was a smaller increase over time in children’s health and development, compared with parents who reported lower positive mental health. These patterns were not evident for the control group. The other investigated variables, i.e., children’s gender, number of children, parents’ country of birth, and parents’ educational level, were not found to moderate the CHD outcome over time.

**Table 4:** **Mixed linear model estimates for moderating effects on Child Health and Development (CHD)**

Variables	β	SE	*t*	95% CI	*p*-value
Child age × Time × (I)	0.52	0.24	2.14	0.04, 1.00	.033
Child age × Time × (C)	0.50	0.26	1.94	-0.01, 1.01	.053
Child gender(boy) × Time × (I)	0.19	1.32	0.14	-2.40, 2.78	.885
Child gender(boy) × Time × (C)	1.36	1.34	1.02	-1.27, 3.98	.310
Parents’ birth country (foreign-born) × Time × (I)	0.17	1.67	0.10	-3.10, 3.44	.918
Parents’ birth country (foreign-born) × Time × (C)	-0.27	1.58	-0.17	-3.39, 2.84	.863
Parental education (university) × Time × (I)	0.59	1.31	0.45	-1.99, 3.17	.653
Parental education (university) × Time × (C)	-0.00	1.33	-0.00	-2.62, 2.61	.998
Parental positive mental health × Time × (I)	-0.92	0.23	-3.93	-1.38, -0.46	.000
Parental positive mental health × Time × (C)	-0.41	0.25	-1.64	-0.89, 0.08	.102
Number of children^a^ × Time × (I)	1.91	1.92	1.00	-1.86, 5.68	.320
Number of children^a^ × Time × (C)	3.56	1.81	1.96	-0.01, 7.12	.050

Note. *(I)* = Intervention group; *(C)* = Control group.

^a^Having more than one child.

## Discussion

The purpose of this study was to evaluate the effects of ABC, a universally offered parenting program, using a randomized waitlist-controlled trial. An intervention effect was found for parental self-efficacy, meaning that intervention-group parents reported that their self-efficacy was strengthened. The same result was found for parental perceptions of the children’s health, which also improved over time. The effect sizes were moderate. Regarding moderators, one variable moderated both outcomes, namely, parents’ positive mental health. Parents who rated their mental health more poorly at baseline showed a greater increase in their parental self-efficacy over time, as well as in their children’s health and development. This result indicates that the program may have a greater effect on parents reporting lower mental health at baseline. Further, parents’ educational level and number of children also moderated the PSE outcome, indicating that the program may have a greater effect on self-efficacy for parents who have finished a university education, and for parents with more than one child. Additionally, child age had a moderating effect on CHD, indicating that the program effect might be greater on children’s health and development for parents with older children.

It is notable that parents who rated their mental health lower at baseline showed a greater increase in both the outcome measures. This finding is of importance to the public health aim of diminishing the gap in health. The program also had greater impact on parents with a university education, a finding which has also been identified in a previous study [10]. This could, however, increase the gap in health that is due to the association between education and health, i.e., well-educated individuals report better health [33]. The findings concerning the potential impact of moderators, such as number of children and child age, may also be valuable factors to consider for future program developers in the field of universal parenting programs.

Previous research on universal parenting programs shows somewhat similar results regarding parental outcomes. Reedtz and colleagues [11] reported increases in positive parenting and in parental sense of competence. Similarly, parents who participated in the program 123Magic rated their self-efficacy as higher after the program, even though this study lacked a control group [34]. In contrast to our study, Simkiss and colleagues [16] found no effects related to the children’s well-being. Other evaluations have found effects on child variables, for example, a reduction in child behavior problems [11, 12, 14, 15]. However, these studies rely on problem-oriented measures for evaluating child outcomes, such as the Eyberg Child Behavior Inventory [35], the Child Behavior Checklist [36], and the Strength and Difficulties Questionnaire [37]. Thus, our study results are not comparable on this topic, as we intended to measure the child outcome within a health-promotion perspective.

Regarding moderating effects on the outcome, Sherr and colleagues [10] found that depression had a moderating effect on child management, where parents with higher depression scores obtained a greater effect. This could be considered as being in line with our results, where parents who rated their mental health more poorly at baseline benefited more. A possible explanation for this could be that these parents experience lower self-confidence and thereby have the potential to benefit more from the program. Similar to the study by Sherr and colleagues [10], we also found that having a high educational level positively moderated the outcome, in our case, parental self-efficacy. Previous research on self-efficacy has also shown that having several children is associated with higher self-efficacy [19], and in our trial, the number of children was confirmed to moderate self-efficacy. Gardner and colleagues [18] presented child age as a moderator, where younger children progressed more poorly, a finding which was replicated in our trial. Other studies, such as Ogden and Hagen’s [38], have however found the opposite effect—that is, younger children benefited more from a parenting program. Sanders [17] has stated that the essential principles of positive parenting are cross-culturally robust. This would be in line with the non-significant result of country of birth as a moderator in our study. The present study failed to replicate previous findings on the moderating effect of child gender [18] as we found no differences in effect depending on gender.

### Strengths

Strengths of the study include the measurement of positive outcomes. To our knowledge, there is to date no other evaluation of a universal parenting program that includes only positive outcomes, which is highly relevant in evaluations of a health-promotion program. The focus in previous research has been on the reduction of problem behavior. A problem with using solely problem-oriented outcomes in a general population might be that important improvements in health, well-being, and development are not identified.

A further strength was the independence of the research group from the program developers. Eisner and Humphreys [39] showed that when a conflict of interest was likely for the evaluators of early family interventions and parent-training, greater effect sizes were reported compared with when conflicts of interests were not likely. However, the difference found by Eisner and Humphreys [39] was not significant. Reported reasons for higher effect sizes, in cases where developers are the evaluators as well, are higher program fidelity and pressure to show positive findings [40].

### Limitations

One limitation of our study was the sole reliance of parental ratings. To elaborate on the information based on parental ratings within the trial, a subsample of parents and children was invited to a separate, additional observational study. Parent–child interaction will be explored in a future study, and the observational data will enable us to validate the parental report.

Another limitation within the trial was the difference between parents completing the follow-up measurements and parents who failed to complete them. At both the post- and the follow-up measurements, there were more parents born outside Sweden who failed to respond. A possible reason for this could be language difficulties that impede comprehension and responding. At the baseline measurement, the research staff was present to support parents in completing the questionnaires, whereas subsequent questionnaires were completed by the parents at home. An improvement for future studies could be to offer questionnaires in not only the Swedish language, but also in other common languages of the study population.

Additionally, fewer parents in the intervention group completed the post-measurement compared with parents in the control group. Intervention-group parents who did not complete the post-measurement rated their self-efficacy and children’s health and development higher at baseline compared with intervention-group parents who completed the assessment. The intervention-group parents who did not complete the post-measurement also participated in fewer sessions compared with intervention-group parents who completed the measurement. This could imply that the intervention-group parents who did not complete the post-measurement had less interest in, and need of, the program and were therefore less motivated to continue their participation in the trial.

Furthermore, whereas almost all parents in the intervention group received some parts of the program, only about half of the group participated in all four sessions. This implies that the program could have a greater effect if a larger proportion of the parents had participated in all the sessions. However, parents will always face barriers to participation, such as lack of time, childcare conflicts, and illness. The program participation in this trial therefore probably corresponded well with what can be expected in reality. In a future study of ABC, attendance rate, in relation to program effectiveness, will also be investigated.

A final limitation is the lack of normative data concerning the outcome measures. It is not possible to conclude if the study population was below or above average on the outcome measures (i.e., PSE and CHD) before the start of the trial. For future research, it would therefore be beneficial to possess normative data on these outcomes.

## Conclusions

We found that the ABC program appears to promote parent-rated self-efficacy, as well as parents’ perceptions of child health and development, which indicates that families benefit from parental participation in the ABC program. Additionally, we found that families may benefit differently depending on their baseline characteristics. Having a poor positive mental health, a university-level education, more than one child in the family, and older children, tended to make the families benefit more. Our study thereby adds to an existing understanding of the advantage of offering universal parenting programs as a public health approach to strengthening families. In future, rigorous controlled trials and cost-effectiveness analyses are needed before the ABC program can be classified as effective and cost-effective. Future research is also needed to investigate potential long-term effects of the program, as well as to examine possible mediators.
